# Evaluation of Retinal Histological Alterations Following Carbamazepine Administration in Adult Albino Wistar Rats

**DOI:** 10.7759/cureus.115643

**Published:** 2026-09-02

**Authors:** Shumaila Shakoor, Sabahat Zulfiqar, Rahat Bano Siddiqui, Shamaila Ejaz, Huma Qamar, Muhammad Bahadur Baloch, Talha Laique

**Affiliations:** 1 Anatomy, Allama Iqbal Medical College, Lahore, PAK; 2 Anatomy, Nawaz Sharif Medical College, Gujrat, PAK; 3 Anatomy, Fatima Jinnah Medical University, Lahore, PAK; 4 Anatomy, Lahore Medical and Dental College, Lahore, PAK; 5 Anatomy, CMH Lahore Medical College and Institute of Dentistry, Lahore, PAK; 6 Pharmacology, Lahore Medical and Dental College, Lahore, PAK

**Keywords:** apoptosis, carbamazepine, histology, retinal ganglion cell neuronopathy, wister rats

## Abstract

Background

Epilepsy is characterized by recurrent, unprovoked seizures and is a major global chronic neurological public health concern.

Objective

To evaluate retinal histological alterations, focusing on retinal ganglion cell count and apoptosis, following carbamazepine (CBZ) administration in adult albino Wistar rats. It is an antiepileptic drug mainly used for focal (partial) seizures, where it suppresses excessive neuronal firing by blocking voltage-gated Na⁺ channels.

Study design

A randomized, controlled experimental design was used.

Methodology

In the present study, 18 adult albino Wistar rats were given carbamazepine formulated in alkalinized sodium hydroxide (NaOH) according to their body weight. Following the experimental period, retinal tissues were harvested, fixed, and processed for immunohistochemical analysis using standard staining procedures and DAB (3,3′-diaminobenzidine) visualization. DAB visualization is a method in immunohistochemistry in which the DAB substrate reacts with the enzyme HRP (horseradish peroxidase) to produce an insoluble brown-colored precipitate at the site of the target antigen, allowing microscopic visualization and localization of the protein. Microscopic evaluation was performed to assess retinal ganglion cell density and apoptotic cell count per mm² across retinal layers using an ocular grid under 40X magnification. Animals were randomly divided into three groups (n=6 per group) using the balloting method. Group A was labelled as the control, while Groups B and C were labelled as experimental groups.

Statistical analysis

All the data were processed by SPSS v26.0 (IBM Corp., Armonk, USA). Kruskal-Wallis test was applied to compare means of scores, and one-way ANOVA was applied to compare means of numerical variables of all three groups.

Results

Retinal ganglion cell (RGC) count was observed to be reducing from the control group to experimental Group C. Mean±SD of RGC in Group A was 35.76±3.47, in Group B it was 27.80-±1.47, and in Group C it was 18.63±1.13. Results showed the difference was overall significant (p< 0.001). The control group showed few apoptotic sites, while experimental groups showed enhanced dose-dependent expression, which was moderate in Group B and greatly enhanced in Group C.

Conclusion

We concluded that carbamazepine administration can result in dose-dependent histological alterations in the retina, including retinal ganglion cell loss, layer disorganization, and increased apoptosis, particularly in the retinal pigment epithelium. Further studies are recommended to investigate the underlying mechanisms and determine the long-term retinal safety of carbamazepine, particularly at higher doses.

## Introduction

Epilepsy is a chronic neurological disorder characterized by recurrent, unprovoked seizures and represents a major global public health concern. According to the World Health Organization (WHO), approximately 50 million people are affected worldwide [1]. In Pakistan, the prevalence is estimated at 9.99 per 1000 population, with a higher burden among individuals under 30 years of age [2]. International League Against Epilepsy and the International Bureau for Epilepsy define an epileptic seizure as a transient occurrence of signs and/or symptoms due to abnormal, excessive, or synchronous neuronal activity in the brain [3]. Medications like anti-epileptics, which work by lowering neuronal excitability through modulating voltage-gated sodium channels, are the mainstay of treatment as per literature review [4].

Many conventional anti-epileptics like carbamazepine, phenytoin, phenobarbital, valproic acid, and benzodiazepines are often used because of their effectiveness [5]. Trigeminal neuralgia and mood problems can be treated with carbamazepine (CBZ), a first-line medication for focal seizures. It has a high bioavailability and is metabolized by the liver through hydroxylation and epoxidation [6].

These drugs have several side effects that usually effect visual system, despite their efficacy. Blurred vision, diplopia, and color vision abnormalities are among the visual impairments that have been reported [6,7]. Whether these abnormalities are caused by medications or by epilepsy itself is still a mystery. According to one study, medicine-induced visual dysfunction results from pathological processes like photoreceptor damage and retinal layer disorganization [8]. Carbamazepine has caused visual toxicity, including color vision defects and visual field abnormalities, with occasional reports of retinopathy [9].

Retinal ganglion cells (RGCs), essential for visual signal transmission, may be especially susceptible to drug-induced injury. Long-term drug-related adverse effects (DRAEs*) *have been associated with apoptotic changes in RGCs, possibly due to reduced cerebral and ocular blood flow leading to ischemic damage [8]. Despite reported visual side effects of carbamazepine, experimental evidence regarding its direct histological effects on retinal tissue remains limited. Therefore, this study aimed to evaluate retinal histological alterations, focusing on RGC count and apoptosis, following carbamazepine administration in adult albino Wistar rats.

## Materials and methods

This experimental randomized controlled trial was conducted in the research laboratory of the University of Health Sciences, Pakistan. Eighteen adult male albino Wistar rats, weighing 180-200 g, were obtained from the animal house of the same institution. Animals were housed under controlled environmental conditions (temperature 20 ± 0.5 °C, humidity 50 ± 0.5%, 12-hour light/dark cycle) and provided a standardized diet and water ad libitum. All animals underwent one week of acclimatization prior to the experiment. Ethical approval was obtained from the institutional ethical review committee with ethical approval letter number: UHS/Education/126-17/9031/03-2017. Animals were randomly divided into three groups (n=6 per group) using the balloting method [6]. The balloting method means that the six animals assigned to each group were selected by a simple random procedure to minimize selection bias. Each animal was assigned a unique number, and the numbers were placed on identical slips of paper. The slips were mixed thoroughly, and numbers were drawn blindly to allocate animals sequentially to Groups A, B, and C until each group contained six animals. Duration of treatment was 7 days, and all animals were dissected 24 hours after the last dose.

Group A, as the control group, was given 1 ml of saline alkalinized with 0.1 NaOH thrice daily.

Group B, as the experimental group, was given 50 mg/kg body weight in 3 divided doses of CBZ dissolved in 1ml of saline alkalinized with 0.1 NaOH thrice daily.

Group C, as the experimental group, was given 100 mg/kg body weight in three divided doses of CBZ dissolved in 1 ml of saline alkalinized with 0.1 NaOH thrice daily.

Drug preparation and administration

Carbamazepine powder (C₁₆H₁₂N₂O; molecular weight 236.27 g/mol) was obtained from Sigma-Aldrich (Merck KGaA, Darmstadt, Germany; Cat. No. C4024) and stored at 2-8 °C. The required dose was calculated according to body weight and dissolved in alkalinized 0.1N NaOH (1 ml/kg) [6]. The solution was prepared using a precision analytical balance (Sartorius TE214S; Sartorius AG, Göttingen, Germany) and mixed on a hot plate stirrer (Stuart CB162; Cole-Parmer, Vernon Hills, USA) until a clear solution was obtained.

Tissue processing and immunohistochemistry

Following completion of the experimental period, animals were sacrificed, and eyecups were enucleated. Tissues were fixed in 10% formalin, processed, and embedded in paraffin. Retinal sections of 3 µm thickness were prepared and mounted on saline-coated slides [10].

Thirty-six complete eyeballs from a total of 18 animals were obtained, 12 in each group. The dissection of the eyeball from the bony orbit did not affect the gross anatomy. A twig of the optic nerve was removed from each orbit. Throughout the whole fixing, processing, and paraffin embedding of the eyeball, there was no evidence of tissue shrinkage or loss.

Paraffin-embedded tissue sections were deparaffinized in xylene and rehydrated through a graded ethanol series. Antigen retrieval was subsequently performed by immersing the sections in a phosphate-buffered saline (PBS)-based antigen retrieval solution and heating them in a water bath for 45 minutes. After retrieval, the sections were allowed to cool to room temperature and rinsed with PBS before proceeding with the subsequent staining procedure [6,10]. Endogenous peroxidase activity was blocked with 3% hydrogen peroxide in methanol for 10 minutes. Following washing with PBS, the tissue sections were incubated with Protein Block, Serum-Free (Dako, Agilent Technologies, Santa Clara, USA; Code/Cat. No. X0909) for 30 minutes at room temperature, followed by overnight incubation at 4°C with rabbit polyclonal anti-GFAP primary antibody (Dako, Agilent Technologies, Glostrup, Denmark; Cat. No. Z0334). Subsequently, slides were washed and incubated with polymer enhancer and HRP-conjugated secondary reagent for 30 minutes each. Visualization was achieved using DAB chromogen, followed by hematoxylin counterstaining. Slides were then dehydrated and mounted. Microscopic evaluation of retinal tissue included apoptotic cell count per mm² in all retinal layers and retinal ganglion cell (RGC) count per mm².

Cell counts were performed in five randomly selected fields per slide using an ocular grid under 40X objective magnification on a Leica microscope (Leica Microsystems, Wetzlar, Germany). For assessment of apoptotic expression across the retinal layers, images were examined at both low (4X objective) and high (40X objective) magnifications.

Statistical analysis

Data were analyzed using IBM SPSS Statistics, version 26.0 (IBM Corp., Armonk, USA). Mean±SD was calculated for scores and numeric variables (i.e., RGC distortion, RGC count, and apoptotic cell expression). Kruskal-Wallis test was applied to compare means of scores, and one-way ANOVA was applied to compare means of numerical variables of all three groups. A p-value <0.05 was considered statistically significant. Post Hoc Tukey test was applied to compare difference of mean among groups.

## Results

Histological examination

On light microscopic examination, all layers were easily distinguishable from each other. There was clear demarcation of nuclear layers with plexiform layers. The conventional appearance of the retinal ganglion layer (RGL), composed of healthy and homogeneous RGCs, was seen in the control group, Group A (Fig. 1). The receptor layer was identified by the presence of inner and outer segments of receptor cells. The rods were narrower than the cones, and the distribution varied in peripheral and central parts of the retina.

**Figure 1:**
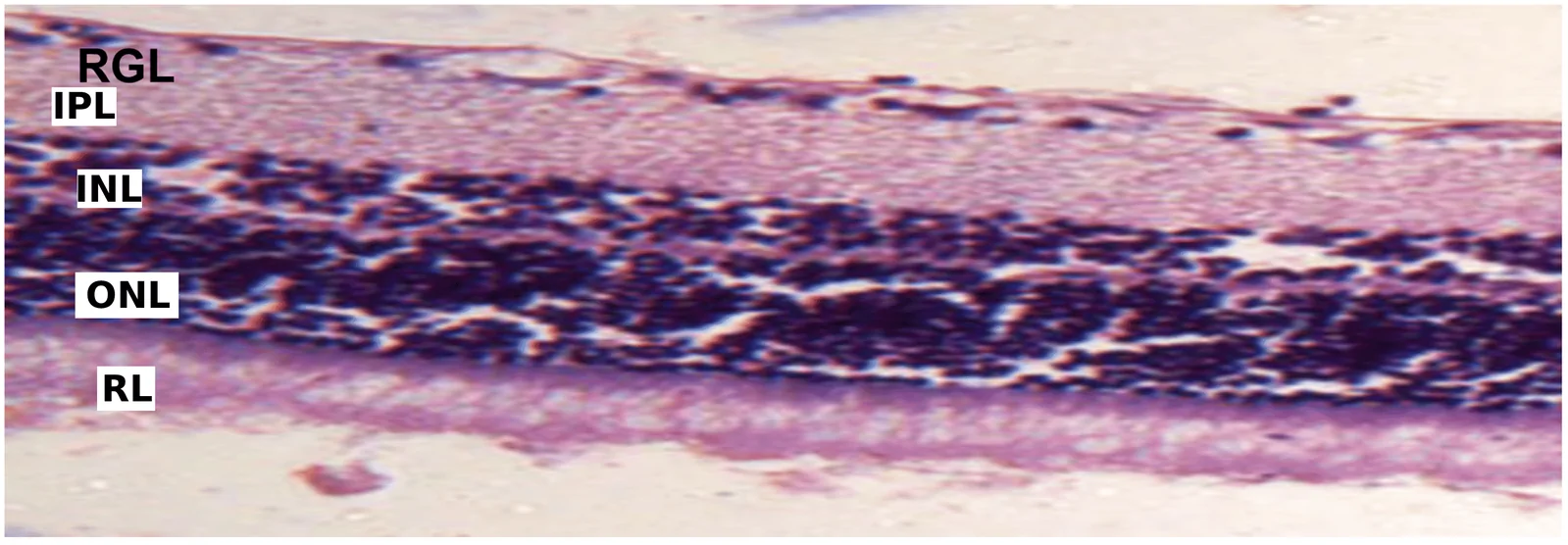
Histological image of retina showing all layers in Group A H&E 10X; N=6; %=100 RGL: retinal ganglion layer, IPL: inner plexiform layer, INL: inner nuclear layer, ONL: outer nuclear layer, RL: receptor layer

Retinal ganglion cell count

Retinal ganglion cells (RGCs) were identified as nuclei present between the nerve fiber layer and the inner plexiform layer (Fig. 2A, 2B, 2C).

**Figure 2:**
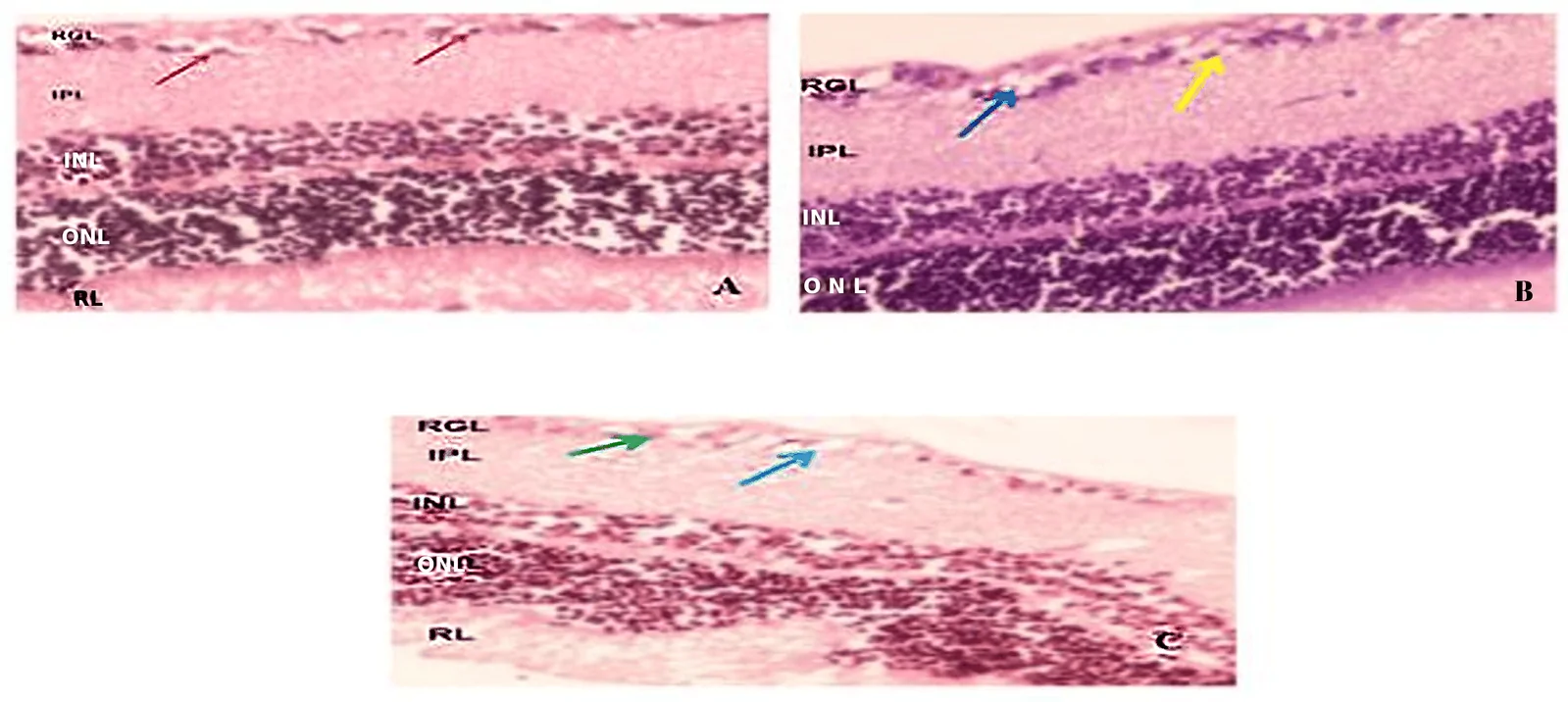
Histological changes for retinal ganglion cell count (A) Control group, Group A: the red arrow shows a ganglion cell with a properly stained nucleus and almost no loss of cells in this layer. (B) Retina of the experimental group, Group B: the ganglion cell count has decreased, with increased empty spaces (blue arrow) and cell nuclei (yellow arrow). (C) Retina of the experimental group, Group C: ganglion cell nucleus in layer (green arrow) and cellular empty spaces/vacuolization (blue arrow). Retinal ganglion cell count was presented as mean±SD, with p<0.05 taken as significant. H&E 40X; N=6 each group; %=100 RGL: retinal ganglionic layer; IPL: inner plexiform layer; INL: inner nuclear layer; OPL: outer plexiform layer; ONL: outer nuclear layer; RL: receptor layer.

Retinal ganglion cell count was observed to be reduced from the control group to the experimental group, Group C. Mean±SD of RGC in Group A was 35.76±3.47, in Group B it was 27.80±1.47, and in Group C it was 18.63±1.13. One-way ANOVA showed the difference was overall significant (p<0.001). Post Hoc Tukey revealed the difference was significant among all three groups (Fig. 3).

**Figure 3:**
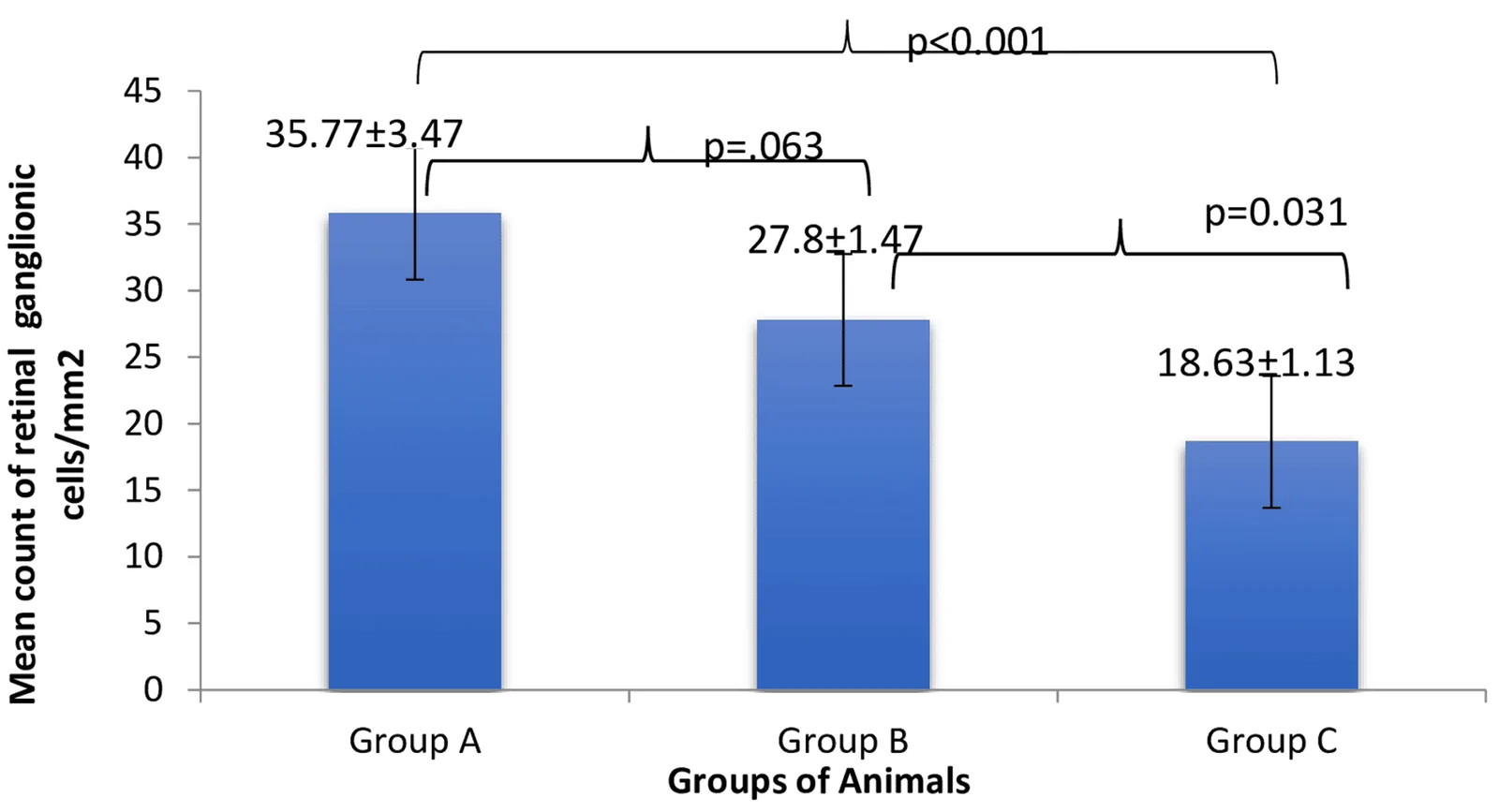
Comparison of number of ganglionic cells among Groups A, B, and C. Error bars showing ± SEM. Overall statistical significance among the groups p<0.001 (as determined by one-way ANOVA with Tukey); N=6 each group; %=100 F-value=170.9; p-value ≤0.05 taken as significant SEM: standard error of the mean

Histological examination of H&E-stained retinal sections revealed morphological changes suggestive of apoptosis, including brownish discoloration and cellular alterations predominantly within the retinal pigment epithelium. However, as no specific apoptotic marker was employed, these findings should be considered apoptosis-like morphological changes rather than definitive evidence of apoptosis (Fig. 4).

**Figure 4:**
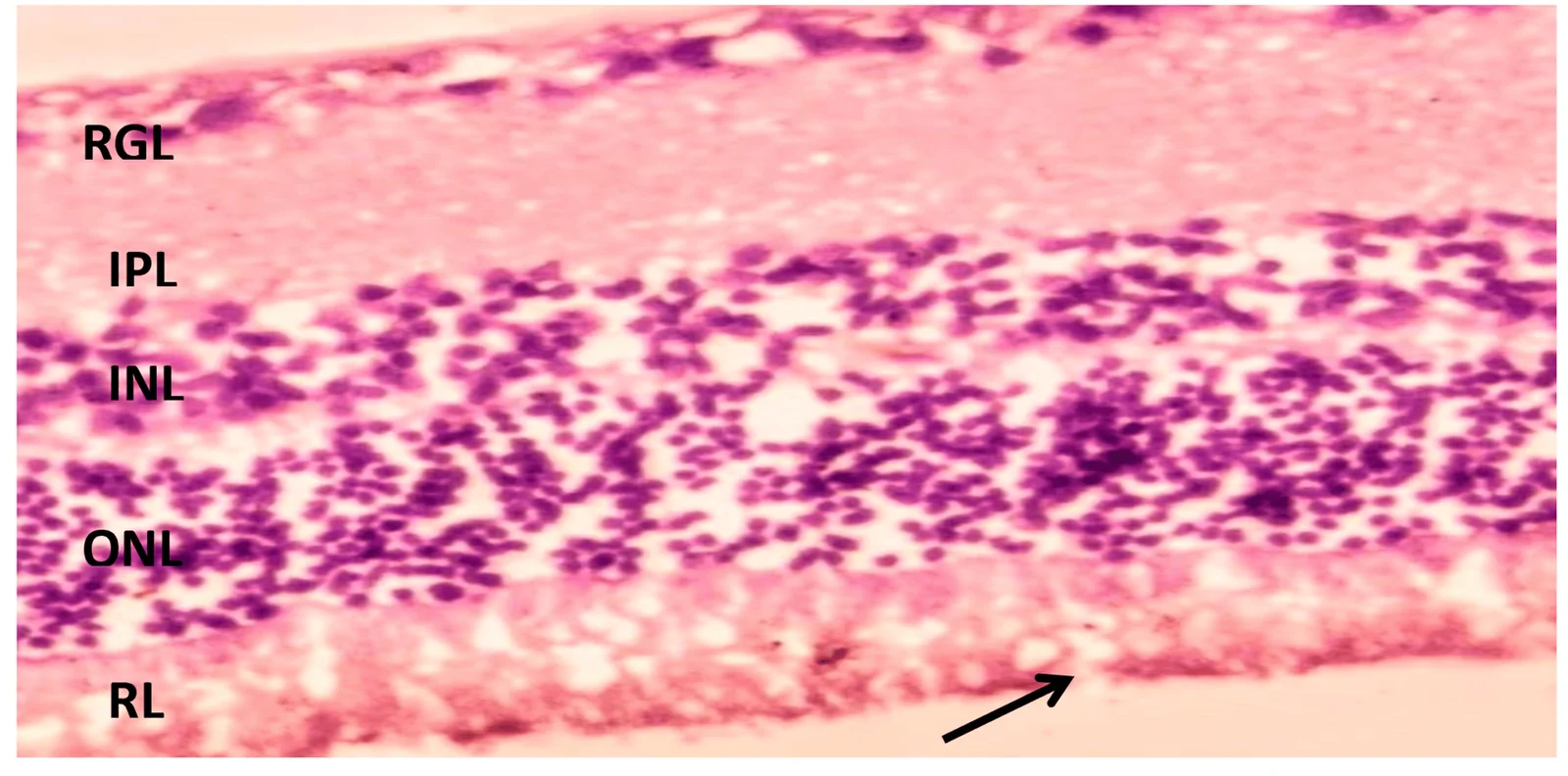
Microscopic image showing apoptosis in pigment epithelium of retina of the control group, Group A (black arrow). Immunihistochemistry H&E 40X; N=6; %=100 Apoptosis of pigment epithelium was presented as mean±SD, with p<0.05 taken as significant. RGL: ganglionic layer; IPL: inner plexiform layer; INL: inner nuclear layer; OPL: outer plexiform layer; ONL: outer nuclear layer; RL: receptor layer

The control group (Group A) showed few apoptotic sites, while the experimental groups showed enhanced, dose-dependent expression, which was moderate in Group B and greatly enhanced in Group C (Fig. 5 and Fig. 6).

**Figure 5:**
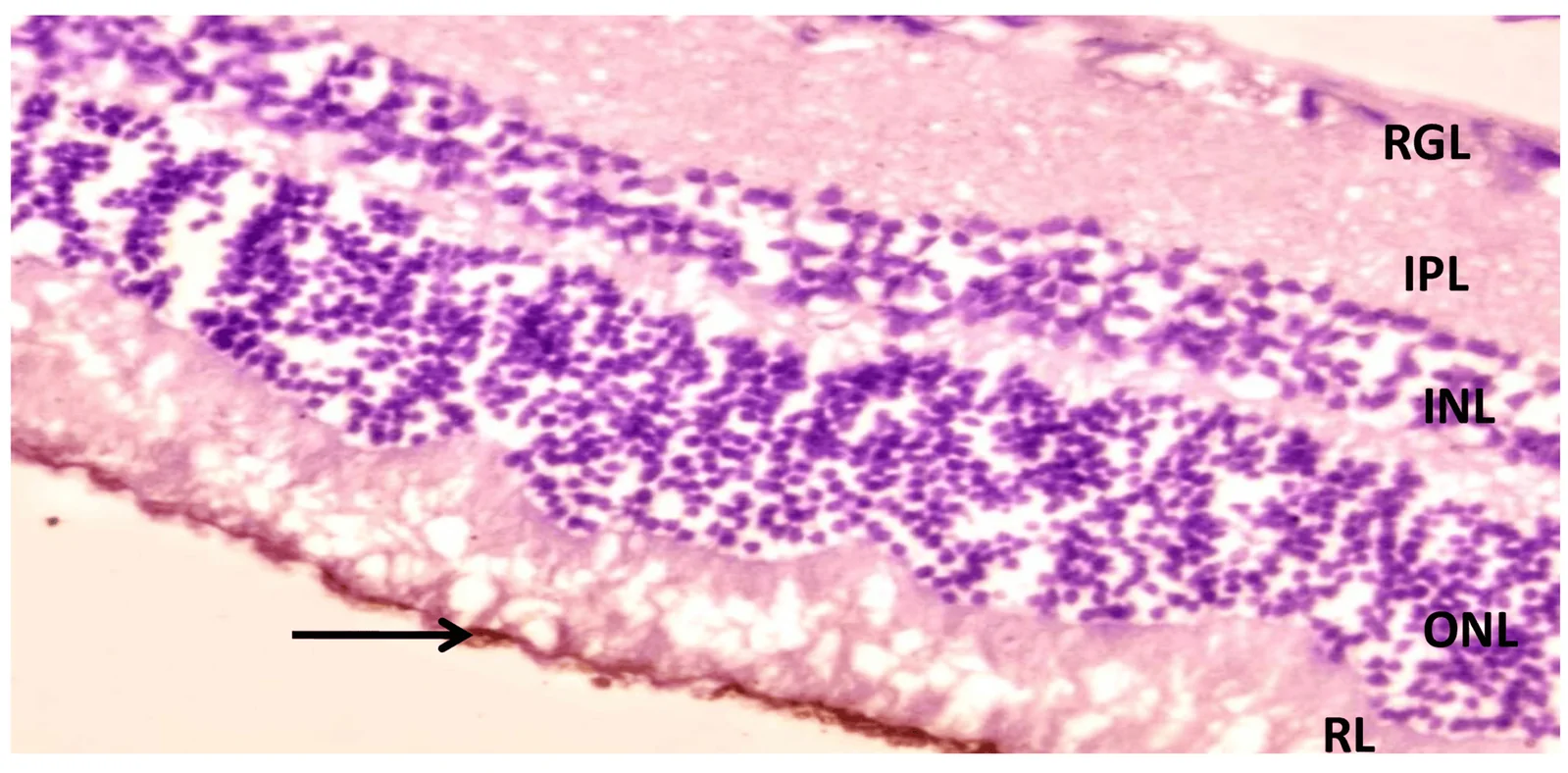
Microscopic image showing apoptosis in pigment epithelium of retina of the experimental group, Group B (black arrow). Immunohistochemistry H&E 40X; N=6; %=100 Apoptosis of pigment epithelium was presented as mean±SD, with p<0.05 taken as significant. RGL: ganglionic layer; IPL: inner plexiform layer; INL: inner nuclear layer; OPL: outer plexiform layer; ONL: outer nuclear layer; RL: receptor layer

**Figure 6:**
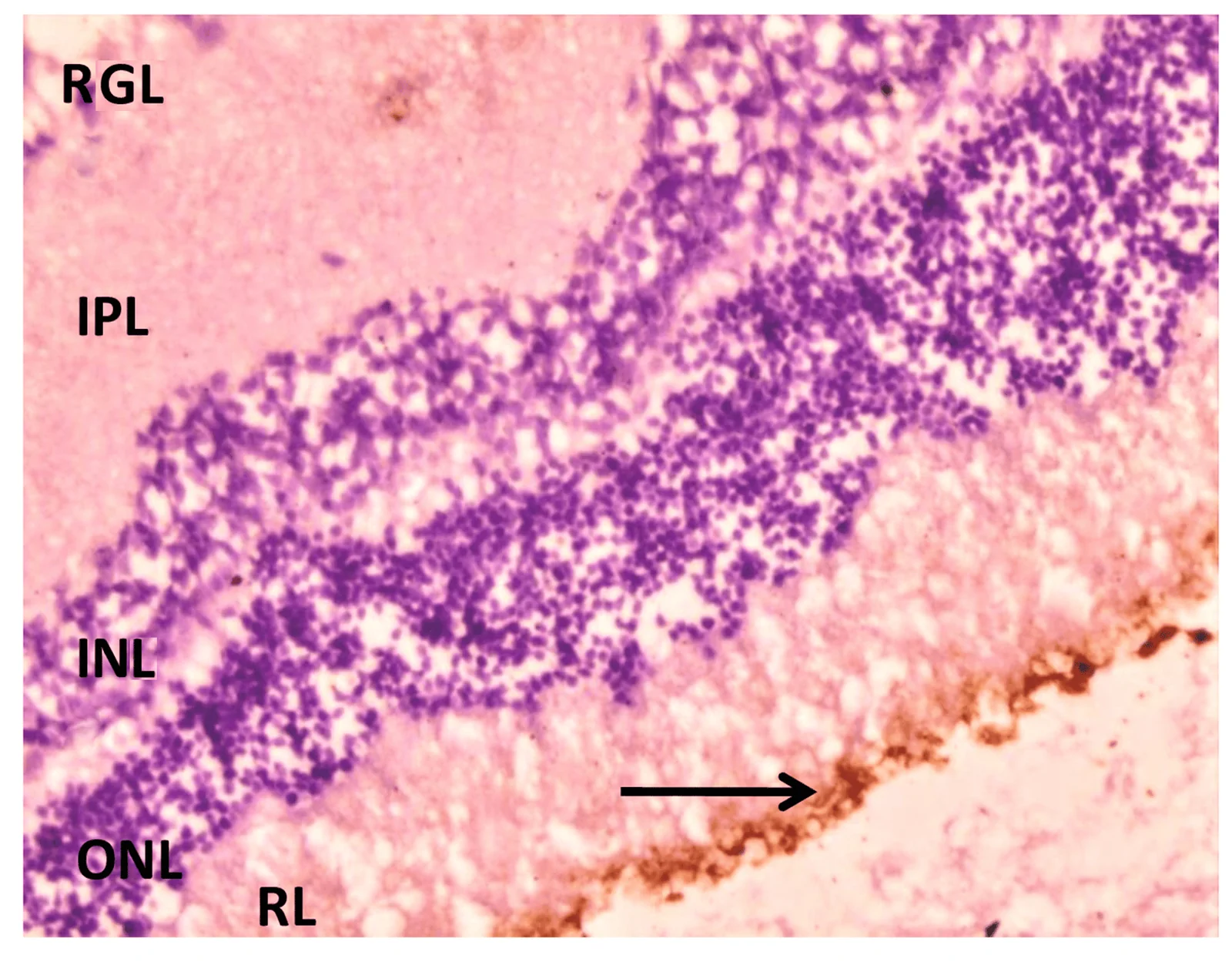
Microscopic image showing apoptosis in pigment epithelium of retina of experimental group, Group C (black arrow). H&E-stained microscopic section showing apoptosis-like morphological changes in the retinal pigment epithelium of the experimental group, Group C (black arrow), at 40X magnification. N=6 RGL: retinal ganglion cell layer; IPL: inner plexiform layer; INL: inner nuclear layer; ONL: outer nuclear layer; RL: receptor layer

The mean count of apoptotic cells in Group A was 2.4±.037, Group B was 9.76±0.79, and Group C was 16.3±1.02. One-way ANOVA showed this difference was highly significant (p<0.001). Post Hoc Tukey revealed significant differences between groups (Fig. 7).

**Figure 7:**
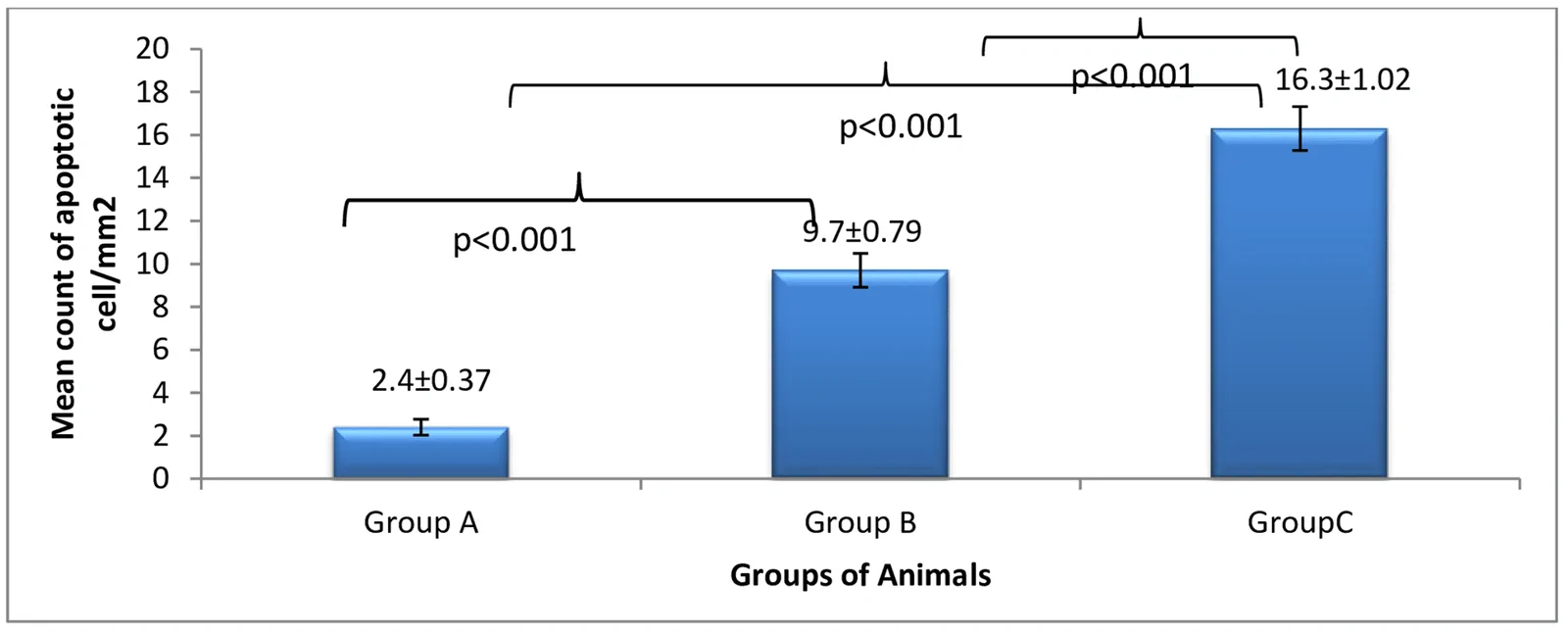
Comparison of mean apoptosis of pigment epithelium among groups A/B/C. Error bars showing ± SEM. Overall significance (p<0.001) (as determined statistically by one-way ANOVA); N=6 each group; %=100 F-value = 1045.04; p-value ≤ 0.05 taken as significant SEM: standard error of the mean

## Discussion

The present study aimed to evaluate the histological effects of carbamazepine (CBZ) on the retina of adult male Wistar albino rats. Male albino Wistar rats were selected to minimize potential variability associated with the female reproductive cycle and fluctuations in ovarian hormones, which may influence physiological and tissue responses to experimental drugs. The use of male animals also provides a more homogeneous experimental population and reduces potential hormonal confounding [11]. The findings demonstrated a dose-dependent reduction in RGC count, along with disorganization of retinal layers and increased apoptotic activity in experimental groups. Immunohistochemical analysis further revealed enhanced apoptotic expression, particularly within the retinal pigment epithelium (RPE).

A significant observation was the reduction in RGC density accompanied by cellular distortion and features suggestive of intracytoplasmic edema. These findings were more noticeable in the higher-dose groups, thus suggesting that CBZ had a dose-dependent neurotoxic effect. According to a previous study, other antiepileptic medications also caused similar changes in retinal neuronal density and structure, thus suggesting that anti-epileptics affect retinal integrity [12].

Its pathophysiology involves excitotoxic and ischemic pathways as underlying mechanisms. According to one study, raised extracellular glutamate levels under hypoxic-ischemic conditions result in excitotoxicity by activating ionotropic glutamate receptors. This causes intracellular calcium excess and damage to neurons [13]. It triggers nitric oxide synthase and other enzymatic cascades that lead to oxidative stress and neuronal death. In retinal neurodegenerative diseases like glaucoma, where RGC loss is a defining characteristic, this mechanism has been thoroughly documented [14]. Secondly, this oxidative stress, either due to an imbalance between pro-oxidant and antioxidant systems or ischemic conditions, exacerbates retinal damage. One major consequence is the generation of free radicals that cause structural damage to retinal layers and reduction in ganglion cell thickness [9]. It has been concluded by previous studies that CBZ impairs ocular perfusion and induces hypoxic conditions, thus promoting oxidative injury that leads to structural disorganization of retinal layers [14].

Additionally, the present project showed elevated apoptotic activity by caspase-3 immuno-reactivity, especially in the RPE. Caspase-3 is a trustworthy indicator of programmed cell death. Previous literature showed that anti-epileptics cause retinal toxicity and apoptosis by decreasing cerebral and ocular blood flow [15]. It has been demonstrated that metabolically active retinal cells, such as RPE and RGCs, are most affected by hypoxia-induced apoptosis.

It is interesting to note that whereas apoptosis was clearly seen in the RPE in this investigation, RGCs showed no discernible apoptotic expression despite having fewer of them. This result implies that rather than acute apoptosis, RGC depletion could be caused by other mechanisms such as necrosis or chronic degenerative processes. Experimental retinal studies have revealed similar differences between apoptotic markers and structural loss [9,16]. Our findings that showed retinal layer disorganization and vascular congestion support the hypothesis of ischemia-induced injury. Similarly, glutamate neurotransmitter levels along with compromised microcirculation also contributed to these histopathological changes [14]. However, more research is required to establish the exact molecular pathways underlying CBZ-induced retinal toxicity.

This study's primary strength is that it used non-epileptic animal models to assess the direct effects of carbamazepine on retinal histology, thereby removing confounding variables linked to the condition. Furthermore, the application of immunohistochemistry allowed for the objective evaluation of cellular apoptotic alterations. The study does have some limitations. Functional visual evaluations were not carried out to link histology results with clinical outcomes. The sample size was small, which added to our limitations. However, long-term research is needed to further understand chronic consequences and the molecular processes underlying retinal damage.

## Conclusions

The present experimental study demonstrated that carbamazepine administration can be associated with dose-dependent histological alterations in the retina of adult albino Wistar rats. A progressive reduction in retinal ganglion cell density was observed with increasing carbamazepine exposure, with the highest-dose group showing the greatest reduction compared with the control group. In addition, increased apoptotic activity was observed in the experimental groups, with more pronounced apoptotic changes at higher doses. Histological examination also demonstrated retinal layer disorganization and alterations involving the retinal pigment epithelium. These findings suggest that prolonged or higher-dose exposure to carbamazepine may be associated with structural and cellular changes in retinal tissue. However, further studies involving larger sample sizes, longer follow-up periods, and functional assessments of visual and retinal function are required to determine the clinical significance and underlying mechanisms of these observed changes.
